# Epimicrobiota Associated with the Decay and Recovery of *Orbicella* Corals Exhibiting Dark Spot Syndrome

**DOI:** 10.3389/fmicb.2016.00893

**Published:** 2016-06-07

**Authors:** Julie L. Meyer, John M. Rodgers, Brian A. Dillard, Valerie J. Paul, Max Teplitski

**Affiliations:** ^1^Soil and Water Science Department, University of Florida–Institute of Food and Agricultural Sciences, Genetics Institute, GainesvilleFL, USA; ^2^Smithsonian Marine Station, Fort PierceFL, USA

**Keywords:** coral disease, coral microbiome, Dark Spot Disease, *Orbicella annularis*, *Orbicella faveolata*, surface mucus layer, Black Band Disease

## Abstract

Dark Spot Syndrome (DSS) is one of the most common diseases of boulder corals in the Caribbean. It presents as sunken brown lesions in coral tissue, which can spread quickly over coral colonies. With this study, we tested the hypothesis that similar to other coral diseases, DSS is a dysbiosis characterized by global shifts in the coral microbiome. Because Black Band Disease (BBD) was sometimes found following DSS lesions, we also tested the hypothesis that DSS is a precursor of BBD. To track disease initiation and progression 24 coral colonies were tagged. Of them five *Orbicella annularis* corals and three *O. faveolata* corals exhibited DSS lesions at tagging. Microbiota of lesions and apparently healthy tissues from DSS-affected corals over the course of 18 months were collected. Final visual assessment showed that five of eight corals incurred substantial tissue loss while two corals remained stable and one appeared to recover from DSS lesions. Illumina sequencing of the V6 region of bacterial 16S rRNA genes demonstrated no significant differences in bacterial community composition associated with healthy tissue or DSS lesions. The epimicrobiomes of both healthy tissue and DSS lesions contained high relative abundances of Operational Taxonomic Units assigned to *Halomonas*, an unclassified gammaproteobacterial genus, *Moritella*, an unclassified Rhodobacteraceae genus, *Renibacterium*, *Pseudomonas*, and *Acinetobacter*. The relative abundance of bacterial taxa was not significantly different between samples when grouped by tissue type (healthy tissue vs. DSS lesion), coral species, collection month, or the overall outcome of DSS-affected corals (substantial tissue loss vs. stable/recovered). Two of the tagged corals with substantial tissue loss also developed BBD during the 18-month sampling period. The bacterial community of the BBD layer was distinct from both healthy tissue and DSS lesions, with high relative abundances of the presumed BBD pathogen *Roseofilum reptotaenium* and an unclassified Bacteroidales genus, similar to previous results. *Roseofilum* was detected in all samples from this study, with the highest relative abundance in healthy tissue from DSS-affected corals sampled in August, suggesting that while DSS is not a precursor to BBD, DSS-affected corals are in a weakened state and therefore more susceptible to additional infections.

## Introduction

It is now clear that many, if not all, animals and plants depend on their microbiota for nutrient acquisition and/or assimilation, responses to environmental stressors, resistance to pathogens, and overall health (Bosch and McFall-Ngai, 2011). Destabilization of the coral epimicrobiota or its invasion by opportunistic pathogens has been linked to a number of coral diseases (Bourne et al., 2009). Despite several decades of coral disease research and the description of over a dozen coral diseases and/or syndromes, pathogens associated with these anomalies have been assigned to less than half a dozen coral diseases (Rosenberg and Ben-Haim, 2002; Lesser et al., 2007; Bourne et al., 2009). In the absence of the causative agent, some coral diseases can be classified as dysbioses, characterized by global changes in the composition, structure, and function of the microbiome. The loss of Oceanospirillales and other abundant members of the commensal microbiota has been linked to the appearance of coral disease symptoms (Cardenas et al., 2012; Vezzulli et al., 2013; Meyer et al., 2014), however, it is unclear whether the dysbiosis is the cause or consequence of the host’s degraded health state.

Dark Spot Syndrome (DSS), also known as “Dark Spot Disease”, is the most prevalent coral pathology in the Caribbean, especially on *Siderastrea siderea* corals. Field studies report that up to 3% of *S. siderea* colonies in relatively pristine reefs in the Bahamas and up to 80% of corals in more impacted reefs have been affected by this disease (Cervino et al., 2001; Borger, 2003; Gochfeld et al., 2006; Voss and Richardson, 2006; Porter et al., 2011). In some locations, DSS also affects other boulder corals, such as *Orbicella* (formerly *Montastraea*) *annularis* and *Stephanocoenia michelenii*, impacting between 3 and 46% of coral colonies (Cervino et al., 2001; Porter et al., 2011). DSS is characterized by a brown, sunken appearance of the tissue, which has been observed spreading over coral colonies at a rate of 3–4 cm/month (Cervino et al., 2001). A significant (13–56%) decrease in the number of intracellular algal symbionts (*Symbiodinium* spp.) and their cell division rates have been reported in DSS-affected *Siderastrea* and *Stephanocoenia* coral tissue (Cervino et al., 2001). Previous work has also shown that the diversity of *Symbiodinium* spp. is lower in DSS-affected polyps than in asymptomatic corals and that certain *Symbiodiunium* genotypes are less abundant in the disease samples (Correa et al., 2009). Despite the high prevalence of DSS in Caribbean corals, total colony mortality due to DSS was generally low; however, impacted colonies experienced up to 50% net tissue loss (Porter et al., 2011).

Multiple studies demonstrate the impact of DSS on coral reef ecosystems throughout the Caribbean, yet the causes, transmission potential, and the role of environmental factors on the appearance of lesions have been the subject of much debate. DSS is typically not observed at great depths; however, corals of the most susceptible species are also more rare at these depths (Gil-Agudelo and Garzon-Ferreira, 2001). Previous work has shown that the prevalence and cover of DSS in *Siderastrea* corals is correlated with summer months when surface sea temperatures are highest (Gil-Agudelo and Garzon-Ferreira, 2001; Borger, 2005; Gochfeld et al., 2006), but similar patterns were not detected in DSS-affected *Montastraea* corals (Gil-Agudelo and Garzon-Ferreira, 2001). While lower DSS prevalence was reported in more pristine ecosystems (Voss and Richardson, 2006) compared to sites more heavily impacted by human activities (Cervino et al., 2001; Borger, 2003; Voss and Richardson, 2006; Porter et al., 2011), lesioned corals were not distributed along a gradient of human impact (Gil-Agudelo and Garzon-Ferreira, 2001). Nutrient enrichment also did not increase prevalence of DSS (Gochfeld et al., 2006). Corals exhibiting DSS lesions have been observed both in a clumped fashion (potentially indicative of a colony-to-colony transmission) and in a randomly distributed fashion (Gil-Agudelo and Garzon-Ferreira, 2001; Voss and Richardson, 2006). In a common garden experiment, three out of five colonies displayed signs of the syndrome after 5 months when placed on a reef where ∼50% of the colonies were diseased (Gochfeld et al., 2006). It is not clear, however, whether these colonies developed DSS due to transmission from nearby colonies or because they experienced the same environmental stress, which led to the observed disease symptoms. In controlled aquarium studies no transmission of the disease was observed over 11 days when water-borne or direct contact transmission modes were tested (Randall et al., 2016).

Characterization of DSS lesions using clone libraries revealed differences in the microbial community composition compared to healthy corals and identified *Photobacterium*, two *Vibrio* species (including *V. campbellii*), *Corynebacterium, Acinetobacter*, Parvularculaceae, and *Oscillatoria* as well as a terrestrial phytopathogenic fungus *Rhytisma acernum* as potential culprits of the disease (Sweet et al., 2013). However, Illumina sequencing and PhyloChip microrray analyses did not reveal significant differences in the microbial community composition of DSS lesions and healthy coral epimicrobiomes (Kellogg et al., 2014; Randall et al., 2016). Nevertheless, PhyloChip microarrays detected higher abundance of the cyanobacterium *Pseudoscillatoria corallii* (now classified as *Roseofilum reptotaenium* (Casamatta et al., 2012)) in dark spot lesions than in healthy tissues sampled in multiple locations. However, these differences were not statistically significant (Kellogg et al., 2014).

Because *R. reptotaenium* has been linked to Black Band Disease (BBD) (Rasoulouniriana et al., 2009; Casamatta et al., 2012; Meyer et al., 2016), this observation suggests that the same pathogen may be responsible for both types of the disease signs. In fact, BBD lesions were sometimes observed surrounded by DSS, and approximately 10% of colonies with DSS later developed BBD (Borger, 2005). Therefore, with this study we aimed to test the overall hypothesis that the DSS is a precursor of BBD and that the same pathogen is responsible for the appearance of both lesion types.

## Materials and Methods

### Sample Collection

*Orbicella annularis* and *O. faveolata* corals with signs of DSS were located and tagged at Carrie Bow Cay, Belize while scuba diving at depths up to 20 m in February 2013. This study was conducted in parallel with the characterization of coral microbiome transitions leading to BBD in the same ecosystem over the same period (Meyer et al., 2016). Twenty-four coral colonies were tagged and followed for ∼31 months, approximately 1/3 of the colonies were asymptomatic, 1/3 had DSS and 1/3 had BBD. Of them, eight colonies had dark spot lesions during the 31-mo observation period. Samples of DSS lesions and surface mucus from healthy tissue on lesioned corals were collected by aspiration with needle-less sterile syringes that were uncapped immediately prior to the sample collection as in (Ritchie, 2006). Tagged corals were re-sampled in July 2013 and August 2014, when possible (**Table 1**). Most tagged corals were photographed at the time of sample collections as well as in September 2015. In the shore-side laboratory, samples were spun in a microfuge at 4,000 rpm for 5 min, seawater was decanted, and the pelleted mucus was preserved with approximately 5–10 volumes of RNAlater (Qiagen, Germantown, MD, USA) and stored at -20°C until extraction of nucleic acids. Genomic DNA was extracted with a PowerSoil DNA isolation kit according to the manufacturer’s instructions (MoBio, Carlsbad, CA, USA).

**Table 1:** Summary of coral surface microbiota sampled and the overall health status of the coral colony in August 2014.

Coral	Sample Type	February 2013	July 2013	August 2014	Visual assessment
*Orbicella annularis* Oa26	Dark Spot	x			Substantial tissue loss
	Healthy Tissue	x		x	
*O. annularis* Oa30	Dark Spot	x	x	x	Substantial tissue loss
	Healthy Tissue	x	x	x	
*O. annularis* Oa32	Dark Spot	x	x	x	Substantial tissue loss
	Healthy Tissue	x	x	x	
	Black Band		x		
*O. annularis* Oa42	Dark Spot	x	x	x	Stable
	Healthy Tissue	x	x	x	
*O. annularis* Oa48	Dark Spot	x		x	Stable
	Healthy Tissue			x	
*O. faveolata* Of27	Dark Spot	x		x	Substantial tissue loss
	Healthy Tissue	x		x	
*O. faveolata* Of43	Dark Spot	x		x	Recovered
	Healthy Tissue	x		x	
*O. faveolata* Of50	Dark Spot	x			Substantial tissue loss
	Healthy Tissue	x		x	
	Black Band			x	


### PCR Detection of the *Rhytisma* Fungus

We developed a PCR assay to detect the presence of the fungus *Rhytisma* that was previously identified in DSS lesions from Caribbean corals (Sweet et al., 2013). The newly developed primer, Rhy_ITS_1F (5′-CCGATTCCACCCTTGATG-3′) was used in conjunction with the previously published primer, ITS4 (5′-TCCTCCGCTTATTGATATGC-3′) (White et al., 1990) to specifically amplify partial ribosomal RNA genes and internal transcribed regions of the coral-associated *Rhytisma* (GenBank Accession KC521543). M. Sweet provided samples of DNA extracted from DSS lesions from the published study (Sweet et al., 2013) to be used as positive controls for the assay. Samples were amplified in 30 μl reactions containing 1.7U *Taq* DNA Polymerase with 1X Standard *Taq* Buffer (New England Biolabs, Ipswich, MA, USA), 0.75 μl DMSO, 0.2 mM each dNTP, 0.5 μM primers, and approximately 10 ng of DNA template. The reaction conditions were an initial denaturation at 94°C for 5 min, followed by 35 cycles of 94°C for 30 s, 55°C for 30 s, 72°C for 45 s, and a final elongation at 72°C for 5 min.

### 16S Illumina Tag Sequencing

The V6 region of bacterial 16S rRNA genes was amplified from whole community DNA in triplicate for each sample with previously published primers (Eren et al., 2013). Samples were amplified in 25 μl reactions containing 0.5 Units Phusion High-Fidelty Polymerase (New England Biolabs, Ipswich, MA, USA), 1X Phusion HF Reaction Buffer, 0.75 μl DMSO, and 0.2 mM each dNTP. Triplicate PCR amplifications were pooled for each sample, cleaned with a MinElute kit (Qiagen, Germantown, MD, USA), visualized on an ethidium bromide stained 1% agarose gel, and quantified by NanoDrop (ThermoScientific, NanoDrop Products, Wilmington, DE, USA). Two hundred nanograms of each cleaned amplicon library was submitted to the Genomics Core Facility at Pennsylvania State University where the pool libraries were size selected with a 2% agarose PippinPrep cassette to produce a narrow range of fragment sizes from 200 to 240 bp for sequencing (confirmed by bioanalyzer) and cleaned again to remove agarose. Sequencing was performed on an Illumina MiSeq with a 150-bp paired-end protocol, using single indexing.

Sequencing reads were parsed by Illumina index at the sequencing center. Reads were then further parsed by the inline barcode, paired reads merged, and primers and adaptors removed using a combination of tools in cutadapt (Martin, 2011), Galaxy (Giardine et al., 2005; Blankenberg et al., 2010; Goecks et al., 2010) and eautils (Aronesty, 2011). Parsed, quality-filtered sequencing reads are publicly available through NCBI’s Sequence Read Archive (SRA) under the BioProject ID PRJNA308473. Sample names were added to the definition lines of sequencing reads using sed and concatenated into one fasta file, to make them compatible for analysis in QIIME v1.8 (Caporaso et al., 2010). Two BBD samples included here were part of a previously published study (Meyer et al., 2016), but were re-analyzed in this study, beginning with OTU clustering, as the samples were from coral colonies also displaying DSS. Clustering of Operational Taxonomic Units (OTUs) at 97% similarity was performed with the subsampled open-reference OTU picking method (Rideout et al., 2014), with no removal of singletons. The Greengenes reference dataset version 13.8 (DeSantis et al., 2006) was used as the reference for OTU picking and for taxonomy assignment with uclust (Edgar, 2010). OTUs identified as mitochondrial DNA or as chloroplasts were removed from further analyses. Community structure was analyzed in R with phyloseq (McMurdie and Holmes, 2013) and plotted with ggplot2 (Wickham, 2009). Analysis of similarities (ANOSIM) was performed in R using VEGAN v2.0–8 (Dixon, 2003). Differences in taxonomic profiles were analyzed by Welch’s *t*-test (for two groups) or by ANOVA (for multiple groups) with Tukey–Kramer *post hoc* tests and Benjamini-Hochberg False Discovery Rate, *q*-value filter > 0.05 with STAMP (Parks et al., 2014).

## Results

### Overview of Bacterial Community Structure in Healthy and Diseased Corals

**FIGURE 1:**
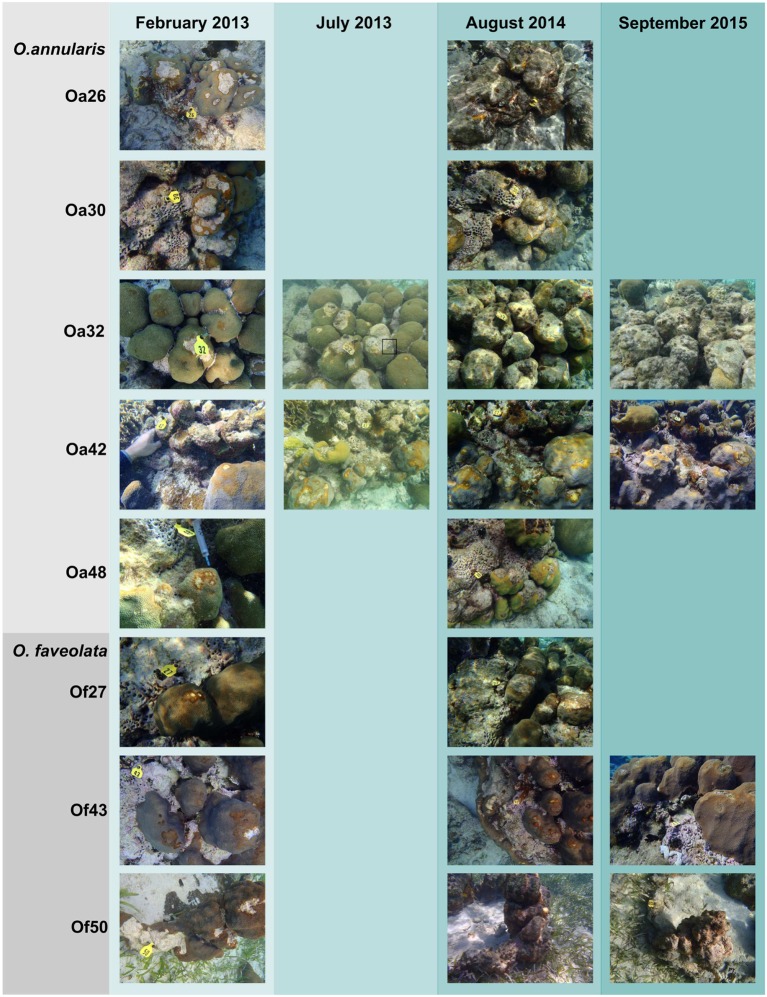
**Photographic time series of *Orbicella* corals affected by Dark Spot Syndrome (DSS).** Photographs from February 2013 to September 2015 of five *Orbicella annularis* and three *O. faveolata* corals at Carrie Bow Cay, Belize. Coral names beginning with Oa are from *O. annularis* and those beginning with Of are from *O. faveolata*. Corals Oa26, Oa30, Oa32, Of27, and Of50 exhibited substantial tissue loss, corals Oa42 and Oa48 remained stable, and coral Of43 appeared to recover from DSS over the observational period. Coral Oa32 had Black Band Disease (BBD) in July 2013, indicated by a black box. Coral Of50 had BBD in August 2014, on the lower right side of the coral colony (not visible in the photograph).

Seventeen samples of *Orbicella* DSS lesions and eighteen samples of healthy tissue on corals with DSS were collected. Between February 2013 and September 2015, substantial tissue loss was observed in five of the eight corals, two corals remained relatively unchanged, and one coral appeared to have recovered from *Orbicella* DSS lesions (**Table 1**; **Figure 1**). Two of the corals that displayed substantial tissue loss also developed BBD during the sampling period. *Rhytisma* ITS sequences were amplifiable in the positive controls, but were not amplifiable from any of the samples collected for the current study.

**FIGURE 2:**
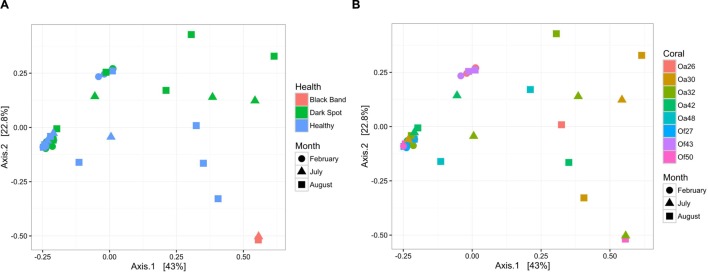
**Bacterial community structure in healthy and diseased coral surface microbiota.** Non-metric multidimensional scaling plot of bacterial community similarity based on Morisita–Horn beta diversity of Illumina MiSeq 16S rRNA gene libraries in the surface mucus layer of corals with DSS. Both healthy tissue and Dark Spot lesions were sampled for each affected coral, with up to three time points sampled (February 2013, July 2013, August 2014). Two of the tagged corals also had BBD at one sampling time. **(A)** The health state of each sample is indicated by color and the collection date is indicated by shape. **(B)** The coral colony (Oa for *Orbicella annularis* and Of for *O. faveolata*) is indicated by color and the collection date is indicated by shape.

**FIGURE 3:**
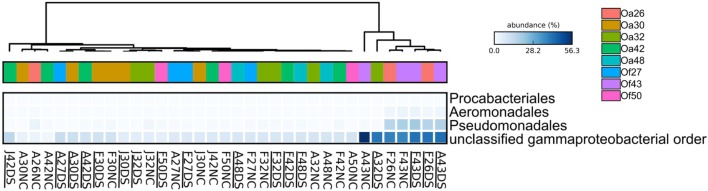
**Heatmap showing the relative abundance of four bacterial orders that varied across *Orbicella* coral colonies.** Coral names beginning with Oa are from *O. annularis* and those beginning with Of are from *O. faveolata*. *O. annularis* coral Oa26 and *O. faveolata* coral Of43 clustered away from the other six coral colonies, due to their higher relative abundance of these four orders. Sample names begin with the first letter of the collection month (F, February 2013, J, July 2013, A, August 2014), followed by the tag number, and end with health status (DS, Dark Spot, NC, healthy tissue). Underlined sample names are the samples of Dark Spot lesions. The intensity of the blue color represents abundance of the indicated bacterial orders.

To characterize bacterial communities, 10,852,271 quality-filtered sequencing reads were analyzed, with 6,576 to 1,029,276 sequencing reads per sample (SRA BioProject ID PRJNA308473). A total of 36,878 OTUs were detected in the 37 surface microbiota samples. The bacterial communities associated with the two BBD samples were similar to each other and distinct from all other samples (**Figure 2A**). In contrast, the bacterial community composition was not significantly different between the healthy tissues of DSS-affected corals and DSS lesions (ANOSIM *R* = 0.016, *p* = 0.217), and no clustering of bacterial communities was observed for either the health condition or the date of sample collection (**Figure 2A**). Excluding the two BBD samples, no significant differences were detected in the abundance of bacterial orders, families, or genera when samples were grouped by health condition (*Orbicella* DSS lesion or healthy tissue), sample date, or the outcome of the infections (substantial tissue loss vs. stable/recovered) (ANOVA with Benjamini–Hochberg False Discovery Rate, *q*-value filter >0.05). No clustering of bacterial communities was observed based on coral species (**Figure 2B**). However, some clustering was observed for a few individual coral colonies such as *O. faveolata* corals Of27 and Of43 (**Figure 2B**), which reflects the stability of the microbiota associated with these two coral colonies. In addition, four bacterial orders were significantly more abundant in some coral colonies (ANOVA with Benjamini-Hochberg False Discovery Rate, *q*-value filter >0.05, effect size >0.6), including the betaproteobacterial order Procabacteriales and three gammaproteobacterial orders: Aeromonadales, Pseudomonadales, and an unclassified gammaproteobacterial order discussed in further detail below. *O. annularis* coral Oa26 and *O. faveolata* coral Of43 clustered away from the other six coral colonies, due to their higher relative abundance of these four orders (**Figure 3**).

### Bacteria Associated with Healthy Tissue, Dark Spot, and Black Band Lesions

**FIGURE 4:**
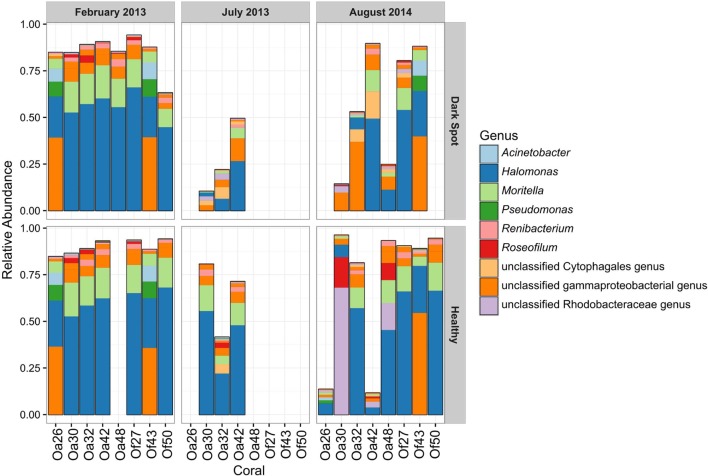
**Relative abundance of the top ten OTUs in healthy tissue and Dark Spot lesions in *Orbicella* corals.** Relative abundance of sequencing reads for the ten most abundant operational taxonomic units (OTUs) in eight tagged corals, across three sampling dates, in healthy tissue and Dark Spot lesions. Coral names beginning with Oa are from *O. annularis* and those beginning with Of are from *O. faveolata*.

The most abundant bacterial genera detected in the healthy and DSS samples were *Halomonas*, an unclassified gammaproteobacterial genus, *Moritella*, an unclassified Rhodobacteraceae genus, *Renibacterium*, *Pseudomonas*, and *Acinetobacter* (**Figure 4**). The ten most abundant OTUs were assigned to these seven genera, as well as to *Roseofilum* and an unclassified Cytophagales genus (**Figure 4**). The unclassified gammaproteobacterial genus was represented primarily by a single OTU that was detected in every sample, and the relative abundance of this OTU was not significantly different between healthy tissue and *Orbicella* DSS lesions or between different sample collection dates. A blastn search of this OTU sequence resulted in equally good hits (100% query coverage with 100% sequence identity) to *Vibrio*, *Serratia*, *Photobacterium*, and *Pantoea* sequences, indicating that the 60-bp V6 region was not able to resolve the taxonomy of this particular OTU. A second OTU assigned to the unclassified gammaproteobacterial genus was also among the ten most abundant OTUs and had a similarly unresolvable taxonomy with perfect matches to *Serratia, Shewanella*, and *Photobacterium.* Rhodobacteraceae appeared to be more abundant in healthy tissues sampled in August 2014, but a two-way ANOVA detected no significant difference in the relative abundance of Rhodobacteraceae related to health and/or sample month. The two BBD consortium samples on DSS-affected corals had high proportions of *Roseofilum*, unclassified genera of Rhodobacteraceae and Bacteroidales, *Fusibacter*, and *Desulfovibrio.* In agreement with the BBD study (Meyer et al., 2016) monitored concurrently with this project, each of the BBD consortium members was detected at lower levels in healthy tissues, as well as in DSS lesions. The relative abundance of *Roseofilum* was not significantly different between healthy tissues and DSS lesions (Welch’s *t*-test, *p* > 0.05). While the two BBD samples were dominated by *R. reptotaenium* (26 and 36% relative abundance), the highest relative abundance of *Roseofilum* in non-BBD samples was detected in two healthy tissue samples, both collected in August 2014 from *O. annularis* corals Oa30 (16%) and Oa48 (9%) (**Figure 4**). Coral Oa30 experienced substantial tissue loss over the study period, but the lesions on Coral Oa48 appeared to remain stable over the study period (**Figure 1**).

### Comparison of the Microbiota of Recovered and Declined Corals

Five of the eight corals in this study showed substantial tissue loss over the sampling period (Oa26, Of27, Oa30, Oa32, and Of50) (**Figure 1**). One coral (Oa32) was well documented with photographs and samples throughout the study period and is representative of the progression of DSS leading to the nearly complete decay of the coral colony. DSS lesions in *O. annularis* coral Oa32 exhibited a loss of the most abundant genus, *Halomonas*, and an increase in the unclassified gammaproteobacterial genus over time (**Figure 4**). The healthy tissue samples in Oa32 retained a high relative abundance of *Halomonas* throughout the study period.

In contrast, only one coral (*O. faveolata* coral Of43) appeared to recover from DSS by September 2015 (**Figure 1**). Coral Of43 sustained high levels of the unclassified gammaproteobacterium throughout the course of the 18-month sampling period, and the overall bacterial community structure did not change over time, as indicated by the clustering of Of43 samples in **Figure 1**. The dominance of this unclassified gammaproteobacterium in all samples from this coral sets it apart from other coral colonies (**Figure 3**). Coral Oa26 clustered with Of43 initially, but over time exhibited a loss of the unclassified gammaproteobacterium and the concurrent decay of the coral colony.

## Discussion

While DSS has been previously reported in boulder corals (Gil-Agudelo and Garzon-Ferreira, 2001), this is the first study to characterize the bacterial community associated with DSS-affected *Orbicella* corals. Based on the sequencing of the V6 hypervariable region of 16S rRNA genes, we detected no shift in the bacterial communities between healthy tissue and *Orbicella* DSS lesions, with all samples containing a high relative abundance of Gammaproteobacteria, especially of the genera *Halomonas*, *Moritella*, and *Pseudomonas*, as well as an unclassifiable gammaproteobacterial genus. These results are consistent with earlier reports (Kellogg et al., 2014; Randall et al., 2016) that detected no significant shift in the microbiome composition of healthy *Siderastrea siderea* corals compared to those with DSS lesions. Sequencing of the V1–V3 hypervariable region of 16S rRNA genes showed no differences in the overall bacterial community composition of healthy or DSS-affected *Siderastrea siderea* corals, no significant differences in bacterial species richness or species diversity, and no significant differences in the relative abundance of putative pathogen taxa (*Vibrio, Corynebacterium, Acinetobacter, Photobacterium, Parvularculacea*, and *Oscillatoria*) (Randall et al., 2016). However, taxon-specific Kruskal–Wallis rank-sum tests identified 9 taxa that were significantly more abundant in diseased corals than in healthy corals and corals experimentally exposed to DSS, including *Alteromonas*, *Aquabacterium*, *Arthrobacter*, *Bermanella*, *Haliscomenobacter*, *Litoreibacter*, Oscillatoriales, *Pseudomonas*, and Sorangiineae (Randall et al., 2016). In contrast, an earlier study using PhyloChip G3 microarrays detected no significant differences in the relative abundance of specific bacterial taxa between healthy *S. siderea* corals and those with dark spot lesions (Kellogg et al., 2014).

In *Stephanocoenia intersepta* corals, the sequencing of clone libraries of bacterial 16S rRNA genes and fungal ITS region revealed differences in the bacterial community composition of healthy corals versus corals with varying sizes of DSS lesions (Sweet et al., 2013). *Burkholderia*, *Pseudomonas*, *Parvibaculum*, and *Ochrobactrum* were detected in all healthy samples, but not in DSS lesions. *Photobacterium* and two *Vibrio* species (including *V. campbellii*) were abundant in lesions and absent from healthy samples. *Corynebacterium*, *Acinetobacter*, Parvularculaceae, and *Oscillatoria* were also present in lesions and absent from healthy corals and increased in abundance in apparently healthy tissue adjacent to lesions (Sweet et al., 2013). A fungal ribotype associated with a terrestrial phytopathogenic fungus, *Rhytisma acernum*, was found in the lesions, but not in healthy samples (Sweet et al., 2013). Our PCR assay did not detect *Rhytisma* in any of the *Orbicella* coral samples in this study, but it did positively amplify *Rhytisma* in the samples of DSS lesions from the *Stephanocoenia intersepta* corals, suggesting that this fungal pathogen is not associated with DSS on different coral types or that *Stephanocoenia* DSS is a different disease than *Orbicella* DSS.

The absence of differences in the microbiomes of apparently healthy tissue or asymptomatic corals and those with DSS in both *Orbicella* (this study) and *Siderastrea* (Kellogg et al., 2014) corals suggests that it is not a dysbiosis syndrome or that DSS may not be the same disease in *Stephanocoenia* corals. The apparent lack of transmissibility of this syndrome supports the hypothesis that it is not infectious disease. In fact, some researchers suggested that DSS is a general stress response, exacerbated by high water temperature or other stressors (Borger, 2005). However, data collected to date does not rule out the possibility that the disease may be caused by a virus that is a normal member of the coral’s virome but under some stress conditions impacts the host’s health. Atypical herpes virus and a megavirus affecting *Symbiodinium* spp. and leading to the loss of algal symbionts have been reported (Correa et al., 2009).

Recovery of corals from DSS has been previously reported, although DSS tended to re-occur on the same corals in 30% of the cases in some studies (Voss and Richardson, 2006). Recovery rates have been reported from 4 to 9% or as high as 30 to 60%, with the differences in the recovery rates likely due to the definition of “recovery” used by the researchers (Borger, 2005; Porter et al., 2011). In some studies, up to 7.1% of colonies regained tissue following disappearance of the lesions, mostly by re-growth over the former lesion (Porter et al., 2011). In this study, three out of eight corals either completely recovered or the spread of dark sport lesions seized. However, the majority of affected corals reported here suffered substantial tissue loss and looked similar to the progression of DSS in *O. annularis* previously documented (Porter et al., 2011).

While we found no differences in the microbiota associated with *Orbicella* DSS lesions and healthy tissue on DSS-affected corals, the BBD layer on two DSS-affected corals hosted a distinct BBD consortium. BBD is caused by *R. reptotaenium*, which is a normal (albeit, minor) member of the microbiota of Caribbean *Orbicella*, *Montastraea*, and *Pseudodiploria* corals (Meyer et al., 2016) and was detected in every sample in this study. Under some conditions, likely associated with an environmental stress, *R. reptotaenium* escapes controls imposed on it by the host and/or other members of the microbiome and overtakes the community, leading to the appearance of BBD. Despite the fact that BBD appeared on 2 out of 8 corals with DSS, there was no evidence that DSS was a precursor of BBD. However, the two corals that developed BBD were among those that experienced the most substantial tissue loss over the observational period. Therefore, we hypothesize that either DSS itself or the environmental conditions leading to it were triggers resulting in the take-over of the host microbiota by *R. reptotaenium.*

## Author Contributions

VP and MT conceived and designed the project. MT collected samples. JM, JR, and BD performed molecular work and data analysis. JM and MT wrote the manuscript and all authors contributed to critical revisions.

## Conflict of Interest Statement

The authors declare that the research was conducted in the absence of any commercial or financial relationships that could be construed as a potential conflict of interest.
